# Oxygen uptake kinetics and ventilatory and metabolic parameters do not differ between moderate‐intensity front crawl and breaststroke swimming

**DOI:** 10.14814/phy2.15361

**Published:** 2022-06-27

**Authors:** Mitch Lomax, Joshua T. Royal, Jernej Kapus, Heather Massey, Zoe Saynor

**Affiliations:** ^1^ School of Sport Health and Exercise Science, University of Portsmouth Portsmouth UK; ^2^ Faculty of Sport University of Ljubljana Ljubljana Slovenia

**Keywords:** flume, muscle metabolism, swimming, $\dot{V}O_{2}$ kinetics

## Abstract

Pulmonary oxygen uptake ($\dot{V}O_{2}$) kinetics have been well studied during land‐based exercise. However, less is known about $\dot{V}O_{2}$ kinetics during swimming exercise and comparisons between strokes is non‐existent. We aimed to characterize and compare the $\dot{V}O_{2}$ kinetics, ventilatory,e and metabolic response to constant velocity moderate‐intensity freely breathing front crawl (FC) and breaststroke (BR) swimming in a swimming flume. These two strokes reflect predominantly upper body versus lower body modes of swimming locomotion, respectively. Eight trained swimmers (4 females, 20 ± 1 years, 1.74 ± 0.06 m; 66.8 ± 6.3 kg) attended 5–6 laboratory‐based swimming sessions. The first two trials determined FC and BR $\dot{V}O_{2\max}$ and the ventilatory threshold (VT), respectively, during progressive intensity swimming to the limit of tolerance. Subsequent trials involved counterbalanced FC and BR transitions from prone floating to constant velocity moderate‐intensity swimming at 80% of the velocity at VT (vVT), separated by 30‐min recovery. Breath‐by‐breath changes in pulmonary gas exchange and ventilation were measured continuously using a snorkel and aquatic metabolic cart system. The ventilatory and metabolic responses were similar (*p* > 0.05) between strokes during maximal velocity swimming, however, vVT and maximal velocity were slower (*p* < 0.05) during BR . During moderate‐intensity swimming, $\dot{V}O_{2}$ kinetics, ventilatory and metabolic parameters were similar (*p* > 0.05) between strokes. In conclusion, when breathing ad libitum, $\dot{V}O_{2}$ kinetics during moderate‐intensity constant velocity swimming, and ventilatory and metabolic responses during moderate‐intensity and maximal velocity swimming, are similar between FC and BR strokes.

## Graphical abstract

The contribution to propulsion from the arms and legs differs between front crawl (FC) and breaststroke (BR) swimming. VO2 kinetics are similar during constant velocity moderate‐intensity FC and BR swimming exercise. Likewise, maximal and submaximal parameters of aerobic fitness are also similar between FC and BR swimming.

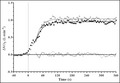

## 1 INTRODUCTION

Pulmonary oxygen uptake ($\dot{V}O_{2}$) kinetics have been widely studied during land‐based exercise to provide insight into the instantaneous rate of energy transfer, substrate utilization and the tolerable duration of exercise (Burnley & Jones, 2007). Only relatively recently have technological advancements led to the development of aquatic‐specific metabolic carts or attachments (e.g., MetaSwim by Cortex, Aquatrainer by Cosmed). This has permitted the examination of $\dot{V}O_{2}$ kinetics during swimming and a more detailed examination of the components of ventilation during this mode of exercise.

Since these developments, studies have compared the impact of exercise intensity (Pelarigo et al., 2017; Sousa et al., 2013, 2014), sex (Reis et al., 2017), fitness level (Reis et al., 2012a), and time‐trial performance (Reis et al., 2012b) on $\dot{V}O_{2}$ kinetics during front crawl (FC) swimming. These studies have greatly elucidated the pulmonary $\dot{V}O_{2}$ kinetic responses to FC in trained swimmers. For example, it has been shown that as swimmers transition from moderate‐intensity exercise (equivalent to 80% of the ventilatory threshold: VT) to heavy‐intensity (Δ25% i.e., VT + 0.25*(maximal $\dot{V}O_{2}$ − VT)) the primary time constant (τ_p_) and primary time delay (TD_p_) shorten or remain unchanged (Reis et al., 2017).

The τ_p_ is limited by the rate of skeletal muscle oxidative metabolism (Barstow et al., 1990; Pringle et al., 2003) and represents the time taken to achieve 63% of the change in $\dot{V}O_{2}$ (Jones & Poole, 2005a). Reis et al. (2012b) have shown that the τ_p_ is positively correlated with the time taken to complete 400 m FC swimming during transitions to both heavy (Δ25%) and severe (Δ70%) exercise. The *τ*_p_ does not appear to differ between male and female swimmers, at least not during moderate‐ and heavy‐intensity FC swimming, although the absolute amplitude of the primary component (*A*_p_) may be higher in males (Reis et al., 2017). Importantly, swimmers who demonstrate a shorter *τ*_p_ and TD_p_ may be better able to minimize the slow component amplitude (*A*_s_) (Pelarigo et al., 2017). This is important as the *A*_s_ reflects an increase in type‐II muscle fiber recruitment and, in turn, enhanced metabolic inefficiency (Jones et al., 2011). It has been shown that an inverse correlation exists between VT and the *A*_s_ meaning that the faster the FC velocity at the VT, the smaller the *A*_s_ will be (Reis et al., 2012a).

To date, however, the investigation of $\dot{V}O_{2}$ kinetics during swimming has focused only on FC; only one other study has examined $\dot{V}O_{2}$ kinetics during BR but they focused solely on the mathematical modeling of the $\dot{V}O_{2}$ slow component (Oliveira et al., 2016). The contribution made to propulsion by the upper body and lower body differs between swimming strokes. Despite being whole‐body strokes, the upper body contributes more than the lower body during FC, but this is reversed during breaststroke (BR) (Bartolomeu et al., 2018; Holmér, 1972). Consequently, FC and BR reflect different modes of locomotion during swimming exercise. Whether or not such differences influence the $\dot{V}O_{2}$ kinetic responses is unknown and warrants further investigation.

The primary aim of the present study was therefore to characterize the $\dot{V}O_{2}$ kinetic response during moderate‐intensity upper (FC) and lower (BR) body dominant modes of swimming locomotion. A secondary aim was to compare the ventilatory and metabolic responses to moderate‐intensity and maximal velocity FC and BR flume swimming. We hypothesized that when a snorkel was used to permit ad libitum breathing, and hence remove the stroke‐induced constraint to breathing, $\dot{V}O_{2}$ kinetics, and the ventilatory and metabolic responses would be similar between FC and BR flume swimming despite the different modes of swimming locomotion.

## 2 MATERIALS AND METHODS

### 2.1 Experimental approach to the problem

Trained swimmers attended the swimming flume laboratory on five or six occasions for experimental testing. Following familiarization with the swimming flume and equipment, the first two trials were used to determine FC and BR‐specific $\dot{V}O_{2\max}$, respectively. The subsequent three or four experimental trials consisted of one FC and one BR constant velocity moderate‐intensity swim per trial, separated by 30 min seated rest. Each swim consisted of a transition from 3 min of prone floating to 6 min of constant work rate exercise at an intensity equivalent to 80% of the velocity at VT (vVT). While six‐minute constant velocity, moderate‐intensity FC, and BR swimming step transitions have little applicability to swimming performance per se, this duration is consistent with that adopted in the wider $\dot{V}O_{2}$ kinetics literature and the choice of strokes permits a comparison between predominantly upper versus lower body modes of swimming locomotion.

All testing took place in a swimming flume (SwimEx, 600‐T, USA) housed in a climatically controlled chamber (water temperature: 29.9 ± 0.3°C; air temperature: 24.0 ± 0.3°C: barometric pressure: 760.8 ± 4.4 mmHg; humidity: 78.8 ± 6.3%). Breath‐by‐breath changes in pulmonary gas exchange and ventilation were measured continuously throughout each trial using a snorkel connected to an aquatic metabolic cart (MetaSwim, Cortext, Germany) with a total dead space (mouthpiece, snorkel, volume flow sensor and splash water protector) of 222 ml. This approach permitted the assessment of stroke‐specific $\dot{V}O_{2\max}$, $\dot{V}O_{2}$ at VT, percentage $\dot{V}O_{2\max}$ at VT, vVT, peak minute ventilation ($\dot{V}$_Epeak_), peak carbon dioxide output ($\dot{V}{\mathrm{CO}}_{2\text{peak}}$), peak respiratory exchange ratio (RER_peak_), peak tidal volume (TV_peak_), and peak *f*_*r*_ (*f*_*r*peak_) values between maximal intensity FC and BR swimming. It also allowed the end‐tidal partial pressures of O_2_ and CO_2_ (PETO_2_ and PETCO_2_, respectively), inspiratory and expiratory time (T_I_ and T_E_, respectively) and the proportion of the total breath cycle time spent in inspiration (T_I_/T_TOT_) to be assessed during maximal swimming. Finally, the constant velocity swims permitted the relevant $\dot{V}$O_2_ kinetic parameters (*τ*_p_, TD_p_, *A*_p_, mean response time [MRT]) and various ventilatory ($\dot{V}$_E_, TV, *f*_*r*_, PETO_2_, and P_ET_CO_2_, T_I_, T_E_, T_I_/T_TOT_) and metabolic ($\dot{V}O_{2}$, $\dot{V}{\mathrm{CO}}_{2}$, RER) parameters to be measured during moderate‐intensity FC and BR swimming. This study was undertaken in accordance with the principles of the Declaration of Helsinki.

### 2.2 Participants

Eight (4 females) trained swimmers (age: 20 ± 1 year; stature: 1.74 ± 0.06 m; body mass: 66.8 ± 6.3 kg) volunteered for this study. All undertook at least 6 h of swim training per week and competed at national university level. All provided fully informed written consent (which also acknowledged that they cannot be identified in the paper and that their data is fully anonymized) and institutional ethics approval was received before the start of the study.

### 2.3 Protocol

#### 2.3.1 $\dot{V}O_{2\max}$ and VT determination

After a separate swimming flume and equipment familiarization session, swimmers completed two progressive velocity swimming tests to the limit of tolerance (T_lim_) to determine $\dot{V}O_{2\max}$ on separate days: 1 FC and 1 BR. Each progressive velocity swimming test was followed by a $\dot{V}O_{2\max}$ verification test 15 min later. Both incremental tests, for the determination of $\dot{V}O_{2\max}$, began with a 3 min baseline period during which participants remained stationary in a prone position in the flume. This was followed by a 3 min warm‐up and then progressive‐intensity swimming test until T_lim_ (starting velocity of 1.00 ± 0.11 m s^−1^ for FC and 0.81 ± 0.04 m s^−1^ for BR). At the end of each 2 min stage, velocity was increased 0.05–0.1 m s^−1^ until T_lim_. Subsequently, swimmers undertook a 5 min cool down at warm‐up velocity, followed by 10 min of seated passive rest outside of the water. Swimmers then completed a supramaximal constant‐velocity test to verify $\dot{V}O_{2\max}$. A 3 min warm‐up preceded a step transition to 105% of the final velocity achieved during the progressive velocity test to T_lim_ (Lomax et al., 2019). The highest 10 s average value achieved during either the $\dot{V}O_{2\max}$ or verification test was taken to represent $\dot{V}O_{2\max}$. $\dot{V}$_Epeak_, $\dot{V}{\mathrm{CO}}_{2\text{peak}}$, RER_peak,_ TV_peak_, and *f*_*r*peak_ were taken from the $\dot{V}O_{2\max}$ or verification test, whichever elicited the highest $\dot{V}O_{2}$. Additionally, PETO_2_, PETCO_2_, T_E_, T_I_, and T_I_/T_TOT_ observed during maximal exercise (i.e., coinciding with $\dot{V}O_{2\max}$) were also reported.

The VT during FC and BR was identified from the respective $\dot{V}O_{2\max}$ tests using the V‐slope method. This was verified using the ventilatory equivalents for O_2_ and CO_2_ and the partial pressure end‐tidal O_2_ and CO_2_ methods by two independent observers trained in the technique (Beaver et al., 1986; Lomax et al., 2019). The VT during FC and BR tests was used to determine the velocity of all subsequent FC and BR 6 min constant velocity swims (Lomax et al., 2019).

#### 2.3.2 Constant velocity swims and analysis of $\dot{V}O_{2}$ kinetics

Swimmers completed 3 or 4 FC and BR constant velocity swimming trials. One FC and 1 BR swim were completed per trial. The order of the swims was counterbalanced between participants and within trials. Each swim consisted of 3 min of prone floating (baseline), 6 min of constant velocity swimming at 80% vVT and 6 min of prone floating (recovery). A 30 min seated rest then separated the end of the first swim and the start of the next swim.

$\dot{V}O_{2}$ from each swim per stroke and per repeat trial was first blinded and a 5‐breath moving average was used to identify outliers: With any breath greater than 2.5 standard deviations from the moving average removed. The remaining $\dot{V}O_{2}$ data per stroke and trial were then linearly interpolated to 1 s, time‐aligned to the start of the 6 min swim (*t* = 0 s) and ensemble averaged. As the cardiodynamic phase is typically 15–20 s in length, the first 15 s from the onset of exercise were visually identified and omitted to remove this phase from analysis (Breese et al., 2019). The phase II monoexponential portion of the $\dot{V}O_{2}$ response was then characterized using the following equation (GraphPad Prism) adapted from Jones and Poole (2005b).

$$\dot{V}O_{2\left(t\right)}=\Delta \dot{V}O_{2A}\left(1-e{-}^{\left(t-\mathrm{TD}/\tau \right)}\right)$$

where $\dot{V}O_{2\left(t\right)}$ is the absolute $\dot{V}O_{2}$ at a given time in s, Δ $\dot{V}O_{2A}$ is the change in $\dot{V}O_{2}$ amplitude from baseline, TD is the time delay in s and *τ* is the time constant.

The MRT was derived to define the overall kinetics during FC and BR by constraining *TD* to 0 s and fitting from the start of the 6 min swim. The entire $\dot{V}O_{2}$ kinetic response was expressed both in absolute terms and relative to $\dot{V}O_{2\max}$ by stroke.

As a $\dot{V}O_{2}$ plateau was observed in the second half of each swim (minutes 3–6), $\dot{V}$_E_, TV, *f*_r_, $\dot{V}CO_{2}$, RER, PETO_2_, PETCO_2_, T_I_, T_E_, and T_I_/T_TOT_ were averaged per stroke. The mean of each swim was then averaged across all trials to give a single value for each stroke. We have shown previously that the test‐re‐test coefficient of variation for $\dot{V}O_{2}$, $\dot{V}{\mathrm{CO}}_{2}$, $\dot{V}$_E_, TV, *f*_*r*_, PETO_2_, and PETCO_2_ during moderate‐intensity FC flume swimming is 2.8%–8.5% (6.2% for $\dot{V}O_{2}$) when using the MetaSwim metabolic cart (Lomax et al., 2019).

### 2.4 Data analysis

Normality of data were assessed using Shapiro–Wilks tests. Paired samples *t*‐tests assessed for differences in all parameters between FC and BR with the exception that Wilcoxon Signed‐Rank tests were used to compare absolute and relative $\dot{V}O_{2\max}$ and vVT during maximal velocity FC and BR swimming, and relative $\dot{V}O_{2}$, *f*_*r*_ and RER during constant velocity swimming. Additionally, Spearman's rho was used to assess for a correlation between $\dot{V}O_{2\max}$ and *τ*_p_ per stroke and Pearson's r to assess for a correlation between *τ*_p_ and maximal velocity per stroke.

Effects sizes were calculated using Cohen's *d* for parametric data with an effect size of 0.2 deemed small, 0.6 moderate, 1.2 large, 2.0 very large and 4.0 extremely large (Hopkins et al., 2009). For non‐parametric data, *r* was used, whereby *r* is the *z* score divided by the square root of the total number of observations. A value of 0.1 was deemed small, 0.3 moderate, and 0.5 and above large (Field, 2013). Effect sizes less than small were reported as no effect. Unless otherwise stated, data are presented as mean and standard deviation (SD).

## 3 RESULTS

$\dot{V}O_{2\max}$, $\dot{V}O_{2}$ at VT, percentage of $\dot{V}O_{2\max}$ at VT, $\dot{V}$_Epeak_, $\dot{V}{\mathrm{CO}}_{2\text{peak}}$. RER_peak_, and *f*_*r*peak_ were similar (*p* > 0.05) between maximal velocity FC and BR. Similarly, PETO_2_, PETCO_2_, T_E_, T_I_ and T_I_/T_TOT_ at maximal exercise were similar (*p* > 0.05) between strokes (Table 1). In contrast, both vVT (*z* = −2.214, *p* = 0.03) and maximal velocity (*t* = 7.000, *p* < 0.001) were faster in FC (Table 1).

**TABLE 1:** Ventilatory and metabolic data in response to maximal FC and BR swimming: Group mean ± SD

Variable	FC	BR	Effect size
$\dot{V}O_{2\max}$ (L min^‐1^)	3.78 ± 0.89	3.36 ± 0.78	Small effect
$\dot{V}O_{2\max}$ (ml^−1^ kg^−1^ min^−1^)	54.12 ± 13.37	50.62 ± 12.20	Small effect
$\dot{V}O_{2}$ at VT (L min^−1^)	1.97 ± 0.83	1.90 ± 0.59	No effect
Percentage $\dot{V}O_{2\max}$ at VT	53 ± 12	57 ± 8	Small effect
vVT (m s^−1^)	1.18 ± 0.20	0.91 ± 0.04*	Large effect
Maximal velocity (m s^−1^)	1.60 ± 0.13	1.35 ± 0.14**	Very large
$\dot{V}{\mathrm{CO}}_{2\text{peak}}$ (L min^−1^)	3.97 ± 0.94	3.42 ± 0.96	Moderate effect
$\dot{V}$_Epeak_ (L min^−1^)	103.8 ± 21.7	88.9 ± 24.5	Moderate effect
TV_peak_ (L)	2.54 ± 0.51	2.23 ± 0.37	Small effect
*f*_*r*peak_ (breaths min^−1^)	46 ± 9	44 ± 7	Small effect
RER_peak_	1.11 ± 0.09	1.14 ± 0.13	Small effect
PETO_2_ (mmHg)	110.33 ± 4.48	109.18 ± 6.31	Small effect
PETCO_2_ (mmHg)	39.73 ± 2.92	41.71 ± 4.80	Small effect
T_I_ (s)	0.80 ± 0.19	0.90 ± 0.25	Small effect
T_E_ (s)	0.60 ± 0.11	0.68 ± 0.17	Small effect
T_I_/T_TOT_ (%)	57 ± 5	57 ± 10	No effect

Abbreviations: BR, breaststroke; FC, front crawl; PETCO_2_, end‐tidal partial pressures of CO_2_; PETO_2_, end‐tidal partial pressures of O_2_; RER_peak_, peak respiratory exchange ratio; T_E_, expiratory time; T_I_, inspiratory time; TV_peak_, peak tidal volume, *f*_*r*peak_, peak *f*_*r*_; $\dot{V}{\mathrm{CO}}_{2\text{peak}}$, peak carbon dioxide output; $\dot{V}$_Epeak_, peak minute ventilation; VT, ventilatory threshold; vVT, velocity at VT.

* *p* ≤ 0.05 different to FC

** *p* ≤ 0.01.

The $\dot{V}O_{2\max}$ verification tests did not (*p* > 0.05) result in higher $\dot{V}O_{2\max}$ values (FC: 3.48 ± 0.64 L min^−1^; BR: 3.26 ± 0.86 L min^−1^) compared with the progressive velocity swimming tests to T_lim_ (FC: 3.47 ± 0.64 L min^−1^; BR: 3.36 ± 0.82 L min^−1^).

**FIGURE 1:**
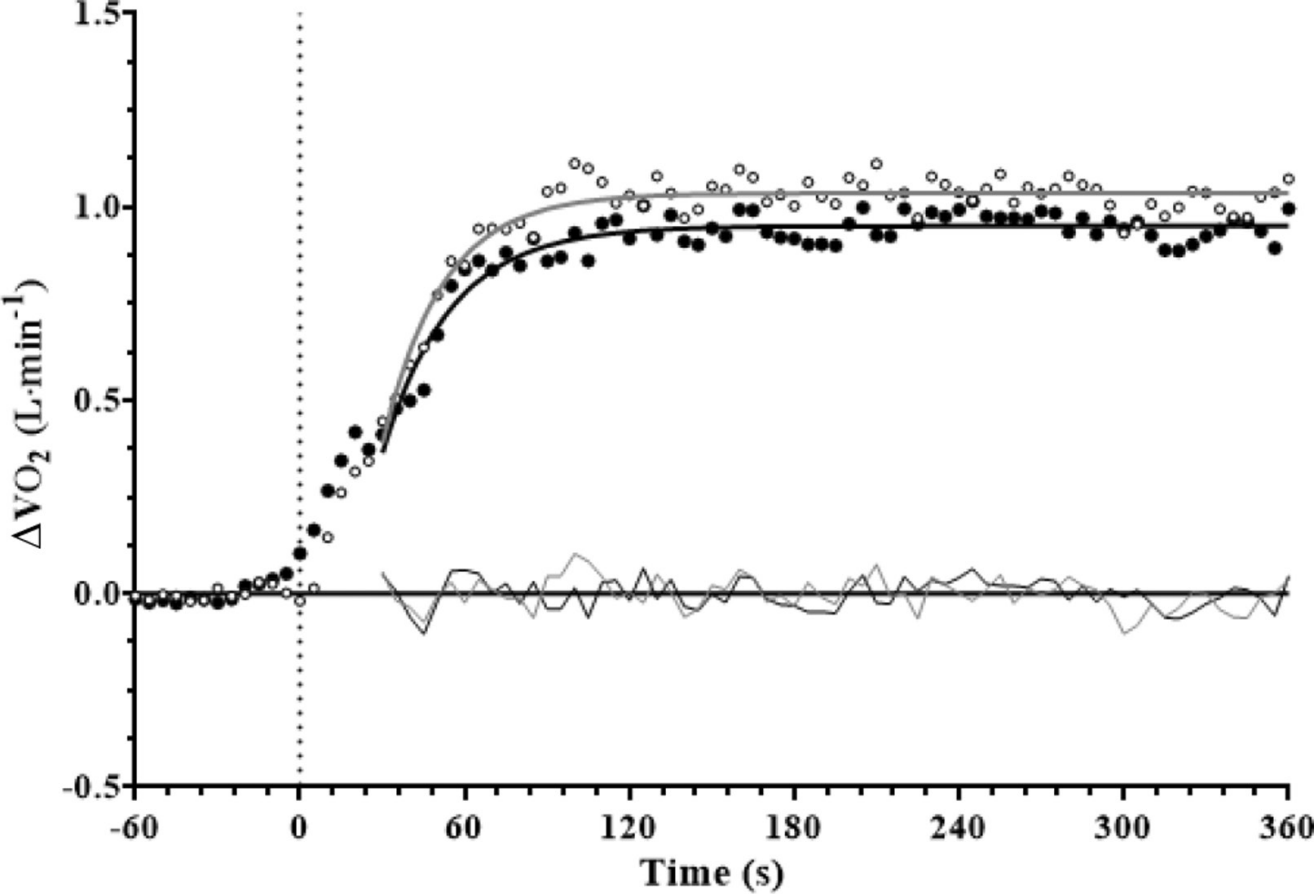
Baseline corrected group mean $\dot{V}O_{2}$ response during FC (open circles) and BR (filled circles) constant velocity swimming. Residuals are also shown.

τ_p_, TD_p_, *A*_p_ and MRT were similar (*p* > 0.05) during moderate‐intensity FC and BR swimming (Table 2; Figure 1). There were no correlations between $\dot{V}O_{2\max}$ and τ_p_ (FC: rho = −0.024, *p* = 0.955; BR: rho = −0.381, *p* = 0.352) or between τ_p_ and maximal velocity (FC: *r* = −0.287, *p* = 0.491; BR: *r* = −0.481, *p* = 0.227) for either stroke. Likewise, all other ventilatory and metabolic parameters were similar (*p* > 0.05) between moderate‐intensity FC and BR swimming (Table 2).

**TABLE 2:** Pulmonary $\dot{V}O_{2}$ kinetics and ventilatory and metabolic data in response to constant velocity moderate‐intensity FC and BR swimming: Group mean ± SD

Variable	FC	BR	Effect size
Baseline $\dot{V}O_{2}$ (L min^−1^)	0.42 ± 0.13	0.51 ± 0.27	Small/moderate
Exercise $\dot{V}O_{2}$ (L min^−1^)	1.38 ± 0.49	1.47 ± 0.35	Small effect
Exercise $\dot{V}O_{2}$ (ml^−1^ kg^−1^ min^−1^)	20.53 ± 6.73	22.13 ± 5.50	Small effect
τ_p_ (s)	24.44 ± 7.30	27.37 ± 9.12	Small/moderate
TD_p_ (s)	18.33 ± 4.98	16.27 ± 7.01	Small effect
*A*_p_ (L min^−1^)	1.04 ± 0.43	0.96 ± 0.22	Small effect
MRT (s)	41.63 ± 8.58	39.94 ± 7.73	Small effect
$\dot{V}O_{2}$ (L min^−1^)	1.22 ± 0.43	1.26 ± 0.33	No effect
$\dot{V}$_E_ (L min^−1^)	32.0 ± 10.3	34.8 ± 8.3	Small effect
TV (L)	1.87 ± 0.29	1.83 ± 0.25	No effect
*f*_*r*_ (breaths min^−1^)	18 ± 5	20 ± 5	Small effect
RER	0.89 ± 0.04	0.86 ± 0.05	Moderate effect
PETO_2_ (mmHg)	102.28 ± 4.70	101.48 ± 7.20	No effect
PETCO_2_ (mmHg)	40.60 ± 2.79	40.03 ± 4.37	No effect
T_E_ (s)	1.54 ± 0.52	1.48 ± 0.49	No effect
T_I_ (s)	2.47 ± 0.97	1.99 ± 0.81	No effect
T_I_/T_TOT_ (%)	59 ± 13	54 ± 13	Small effect

Abbreviations: BR, breaststroke; FC, front crawl; MRT, mean response time; PETCO_2_, end‐tidal partial pressures of CO_2_; PETO_2_, end‐tidal partial pressures of O_2_; RER, respiratory exchange ratio; TD_P_, primary time delay; TV, tidal volume; T_E_, expiratory time; T_I_, inspiratory time; $\dot{V}{\mathrm{CO}}_{2}$, peak carbon dioxide output; $\dot{V}$_E_, minute ventilation; VT, ventilatory threshold; vVT, velocity at VT.

## 4 DISCUSSION

As propulsion is achieved predominantly via the upper body in FC and by the lower body in BR (Bartolomeu et al., 2018; Holmér, 1972), these two strokes represent different modes of swimming locomotion. The primary aim of the current study was to characterize and compare the $\dot{V}O_{2}$ kinetic responses during moderate‐intensity swimming locomotion whereby propulsion was achieved by predominantly the upper body (FC) or the lower body (BR). The secondary aim was to compare the ventilatory and metabolic responses to these two modes of locomotion during moderate‐intensity and maximal velocity swimming.

This study is the first to show that when the stroke‐induced differences in *fr* were removed by use of a snorkel, $\dot{V}O_{2}$ kinetics were similar during constant velocity, moderate‐intensity FC, and BR flume swimming in trained swimmers. Similarly, so too were the ventilatory and metabolic parameters during maximal velocity and moderate‐intensity FC and BR flume swimming.

The FC $\dot{V}O_{2\max}$ of our swimmers (54 ml^−1^ kg^−1^ min^−1^) was similar (50–61 ml^−1^ kg^−1^ min^−1^) to that reported by others, but the $\dot{V}O_{2}$ kinetic response during FC was slower in our swimmers (Pelarigo et al., 2017; Reis et al., 2012a, 2012b, 2017; Sousa et al., 2014). For example, our swimmers had a longer τ_p_, TD_p_, and MRT during FC, and a smaller *A*_p_ compared to those undertaking moderate intensity 200, 600 m, and 30 min of FC swimming (Pelarigo et al., 2017; Reis et al., 2017; Sousa et al., 2013). More specifically, when compared to the findings of Reis et al. (2017), who also utilized a swimming velocity equivalent to 80% of VT, the τ_p_ of our swimmers was 9–10 s slower. The vVT was also slower (1.18 m s^−1^ vs. 1.49 m s^−1^) despite similar $\dot{V}O_{2\max}$ values (54 ml^−1^ kg^−1^ min^−1^ vs. 50–58 ml^−1^ kg^−1^ min^−1^), and VT occurred at a much lower percentage of $\dot{V}O_{2\max}$ (53 ± 12% vs. ~76%–78%). As the VT is a more sensitive indicator of aerobic conditioning than $\dot{V}O_{2\max}$ (Edwards et al., 2003), this suggests that our swimmers were less aerobically conditioned than those in the aforementioned studies; our FC τ_p_ data also supports this notion. The slower kinetics of our swimmers could be indicative of less metabolic stability (Grassi et al., 2011).

Prior research has suggested that τ_p_ is inversely correlated with velocity at $\dot{V}O_{2\max}$ during FC swimming but not $\dot{V}O_{2\max}$ (Reis et al., 2012a). The present study did not observe any correlations between τ_p_ and $\dot{V}O_{2\max}$ nor τ_p_ and maximal velocity. Nevertheless, as the τ during moderate‐intensity exercise reflects the time taken to achieve steady‐state (Berger et al., 2006), a longer τ_p_ would indicate slower cardiovascular and muscular adaptations. This means that a greater length of time would elapse before steady state is achieved (Berger et al., 2006; Sousa et al., 2013) and a larger oxygen deficit would occur (Berger et al., 2006; Burnley & Jones, 2007). This increases both the requirement for anaerobic energy and the production of metabolites (Burnley & Jones, 2007). The longer FC τ_p_ observed in the current study therefore, suggests that our swimmers’ cardiovascular and muscular systems ability to adapt to a moderate‐intensity transition was worse than that reported previously, despite similar $\dot{V}O_{2\max}$ values (Reis et al., 2017).

Although we are unable to delineate the mechanism(s) responsible, this may reflect worse metabolic stability in our swimmers. It has been suggested that $\dot{V}O_{2}$ kinetics might be a marker of metabolic stability, which improves with endurance training (Grassi et al., 2011). Unfortunately, neither the current study nor that of Reis et al. (2017) provided data to assess this, but differences in the magnitude of ADP, Pi, and PCr concentration changes in relation to $\dot{V}O_{2}$ could underpin this. For example, metabolic stability would be worse in our swimmers compared to those in the study of Reis et al. (2017) if they experienced a greater fall in PCr concentration and a greater increase in ADP and Pi concentration for a given $\dot{V}O_{2}$. Alternatively, metabolic stability would also be worse if $\dot{V}O_{2}$ was lower but for a similar change in ADP, Pi, and PCr (Grassi et al., 2011). Regardless of the underpinning cause(s), our data indicate that relying solely on $\dot{V}O_{2\max}$ as a means of comparing aerobic function between swimmers is limited. If the integrated capacity to transport and utilize oxygen is of interest, $\dot{V}O_{2}$ kinetics should be included in any evaluation (Burnley & Jones, 2007).

When the stroke‐induced differences in *f*_*r*_ were removed by use of a snorkel, both maximal and submaximal $\dot{V}$_E_, TV, $\dot{V}O_{2}$, $\dot{V}{\mathrm{CO}}_{2}$, RER, T_I_, T_E_, T_I_/T_TOT,_ PETO_2_, PETCO_2_ were similar between the two modes of swimming locomotion. Likewise, τ_p_, TD_p_, *A*_p_, and MRT were similar between moderate‐intensity FC and BR swimming (Table 2). Cerretteli et al. (1977) have previously shown that $\dot{V}O_{2}$ kinetics (time taken to reach 50% of the change from baseline to steady state $\dot{V}O_{2}$) are slower during land‐based, supine arm only exercise than supine leg only exercise, indicating that muscle group usage impacts the $\dot{V}O_{2}$ kinetic responses. However, Cerretelli et al. (1979) went on to show that training specificity is more important than muscle group usage in determining the $\dot{V}O_{2}$ kinetic response. They found that the on‐kinetics (half time of the $\dot{V}O_{2}$ on‐response) in trained swimmers and kayakers were faster during supine arm cranking and slower during supine leg peddling, but this pattern was reversed in trained runners.

Overall, our data indicate that in trained swimmers, $\dot{V}O_{2}$ kinetics are similar during moderate intensity, and hence steady‐state, upper body and lower body dominant modes of swimming locomotion when breathing is ad libitum via the use of a snorkel. Likewise, the ventilatory and metabolic parameters are also similar during both steady‐state and maximal velocity freely breathing FC and BR swimming. However, it should be noted that while BR relies more on the legs for propulsion than the arms, and FC relies more on the arms for propulsion than the legs (Bartolomeu et al., 2018; Holmér, 1972), both strokes do require activation of the arms, legs and other musculature throughout the stroke cycle (Maglischo, 1993; Nuber et al., 1986).

Unfortunately, logistical and time constraints prevented the inclusion of high‐intensity exercise domains and assessment of butterfly and backstroke; this would have required at least an additional 10 trials per swimmer. Further work is therefore required to confirm whether differences in kinetic parameters exist between strokes at faster velocities. Past studies utilizing the FC stroke have observed a slow component during high‐intensity swimming (Pelarigo et al., 2017; Reis et al., 2012a; Sousa et al., 2013, 2014). Given that the slow component is indicative of muscle inefficiency, which will reduce exercise tolerance (Grassi et al., 2015), any stroke differences will have implications for swimming performance.

It should be acknowledged that the use of a snorkel device will add an additional dead space to the breathing circuit compared to a mask or mouthpiece. This means that during inhalation there will be an increase in the amount of re‐inspired CO_2_. Indeed, it has been shown that wearing a full‐face snorkel increases resting PETCO_2_ by between 4 and 7 mmHg (Lisker et al. (2020).

An increase in external dead space volume will compromise alveolar ventilation and cause arterial hypercapnia unless ventilatory compensation occurs (Ward & Whipp, 1980). Ward and Whipp (1980) showed an upward progressive displacement in the $\dot{V}$_E_–$\dot{V}{\mathrm{CO}}_{2}$ relationship during steady‐state incremental cycling exercise as external dead space volume increased from 100 ml through to 1000 ml. McParland et al. (1991) also showed an increase in exercise ventilation with the addition of an external dead space (940 ml). However, they also showed that when ventilation was fixed during both moderate‐intensity (~$\dot{V}$_E_ of 67 L min^−1^) and high‐intensity (~$\dot{V}$_E_ of 120 L min^−1^) cycling exercise, TV increased (moderate‐intensity: 0.24 L; high‐intensity: 0.41 L) and *f*_*r*_ fell (moderate‐intensity: 2 breaths^.^min^−1^; high‐intensity: 5 breaths^.^min^−1^) compared with normal breathing conditions. Thus, an increase in external dead space volume can impact both ventilation and its component parts.

When translating our findings to a pool environment, it should be noted that the biomechanics of swimming in a flume and the fluid mechanics are different to that of a pool (Guignard et al., 2017). For example, in a swimming flume, the flow of water is directed towards the swimmer and originates from the front of the flume. This pushes the upper limbs backwards and might increase the stroke rate (Guignard et al., 2017; Wilson et al., 1998). Additionally, during flume swimming, the non‐propulsive phases are reduced and the propulsive underwater phases are increased, with the latter aimed at maintaining the correct position in the flume (Guignard et al., 2017); interestingly our swimmers commented that it was harder to maintain the correct position in the flume during BR than FC. Nevertheless, we do not believe that these fluid and biomechanical differences significantly affect the applicability of our findings to a pool setting. In support of this, tethered and untethered flume swimming results in similar $\dot{V}O_{2\max}$ and $\dot{V}$_Epeak_ values, as do pool swimming and tethered flume swimming (Bonen et al., 1980).

It is also pertinent to note here that there were no differences (*p* > 0.05) between the highest $\dot{V}O_{2}$ observed during the progressive velocity swimming tests to T_lim_ and the verification tests, regardless of stroke. However, we do still advocate the inclusion of a verification test, as although at a group level there were no differences, some swimmers (three in the case of both FC and BR) did achieve a higher $\dot{V}O_{2}$ during the verification test.

Finally, while terrestrial studies typically identify the intensity domain(s) to be used in $\dot{V}O_{2}$ kinetic studies by using a combination of physiological parameters (e.g., VT, critical power, $\dot{V}O_{2\max}$) identified from a physiological stress profile test, this approach is not consistently adopted in swimming. Reis et al. (2012a, 2012b, 2017) did adopt this approach with moderate (80% VT), heavy (Δ25%) or severe (Δ70%) intensity constant velocity swims undertaken in their studies. However, others have based transition intensities on a single parameter such as $\dot{V}O_{2\max}$, maximal lactate steady state, the individual anaerobic threshold, or on race paced velocity (Pelarigo et al., 2017; Sousa et al., 2013, 2014). It remains to be seen if this is a more appropriate approach to adopt in swimming.

## 5 CONCLUSION

The impact of swimming locomotion on $\dot{V}O_{2}$ kinetics, ventilatory, and metabolic parameters in trained swimmers were examined during FC and BR flume swimming. These two strokes were chosen as they represent predominantly upper body (FC) and lower body (BR) modes of propulsion. Furthermore, the use of a snorkel permitted ad libitum breathing thereby removing any stroke‐induced breathing constraint. In this situation, vVT and maximal velocity are slower during BR. However, all other ventilatory and metabolic parameters, including $\dot{V}O_{2\max}$, are similar between the two modes of locomotion during maximal swimming. Likewise, $\dot{V}O_{2}$ kinetics and both the ventilatory and metabolic responses are similar during fixed velocity moderate‐intensity FC and BR swimming.

## ETHICS STATEMENT

This study received ethical approval from the University of Portsmouth, Science Faculty Ethics Committee.

## FUNDING INFORMATION

There is no direct funding to report for this study.

## CONFLICT OF INTEREST

No conflicts of interest to declare.

## References

[phy215361-bib-0001] Barstow, T. J. , Lamarra, N. , & Whipp, B. J. (1990). Modulation of muscle and pulmonary O_2_ uptakes by circulatory dynamics during exercise. Journal of Applied Physiology, 68, 979–989. 2341363 10.1152/jappl.1990.68.3.979

[phy215361-bib-0002] Bartolomeu, R. F. , Costa, M. J. , & Barbosa, T. M. (2018). Contribution of limbs' actions to the four competitive swimming strokes: A nonlinear approach. Journal of Sports Sciences, 36(16), 1836–1845. 10.1080/02640414.2018.1423608 29318954

[phy215361-bib-0003] Beaver, W. L. , Wasserman, K. , & Whipp, B. J. (1986). A new method for detecting anaerobic threshold by gas exchange. Journal of Applied Physiology, 60, 2020–2027. 3087938 10.1152/jappl.1986.60.6.2020

[phy215361-bib-0004] Berger, N. J. A. , Tolfrey, K. , Williams, A. G. , & Jones, A. M. (2006). Influence of continuous and interval training on oxygen uptake on‐kinetics. Medicine & Science in Sports & Exercise, 38(3), 504–512. 10.1249/01.mss.0000191418.37709.81 16540838

[phy215361-bib-0005] Bonen, A. , Wilson, B. A. , Yarkony, M. , & Belcastro, A. N. (1980). Maximal oxygen uptake during free, tethered, and flume swimming. Journal of Applied Physiology: Respiratory & Environmental Exercise Physiology, 48(2), 232–235. 10.1152/jappl.1980.48.2.232 7364607

[phy215361-bib-0006] Breese, B. C. , Saynor, Z. L. , Barker, A. L. , Armstrong, N. , & Williams, C. A. (2019). Relationship between (non)linear phase II pulmonary oxygen uptake kinetics with skeletal muscle oxygenation and age in 11‐15 year olds. Experimental Physiology, 104, 1929–1941. 10.1113/EP087979 31512297

[phy215361-bib-0007] Burnley, M. , & Jones, A. M. (2007). Oxygen uptake kinetics as a determinant of sports performance. European Journal of Sport Science, 7(2), 63–79. 10.1080/17461390701456148

[phy215361-bib-0008] Cerretelli, P. , Pendergast, D. , Paganelli, W. C. , & Rennie, W. D. (1979). Effects of specific muscle training on $\dot{V}$O_2_ on‐response and early blood lactate. Journal of Applied Physiology: Respiration, Environmental and Exercise Physiology, 47(4), 761–769. 10.1152/jappl.1979.47.4.761 511683

[phy215361-bib-0009] Cerretteli, P. , Shindell, D. , Pendergast, D. P. , di Prampero, P. E. , & Rennie, D. W. (1977). Oxygen uptake transients at the onset and offset of arm and leg work. Respiration Physiology, 30, 81–97. 877453 10.1016/0034-5687(77)90023-8

[phy215361-bib-0010] Edwards, A. M. , Clark, N. , & Macfadyen, A. M. (2003). Lactate and ventilatory thresholds reflect the training status of professional soccer players where maximum aerobic power is unchanged. Journal of Sports Science and Medicine, 2, 23–29. 24616606 PMC3937571

[phy215361-bib-0011] Field, A. F. (2013). Discovering statistics using IBM SPSS statistics. Sage.

[phy215361-bib-0012] Grassi, B. , Porcelli, S. , Salvadego, D. , & Zoladz, J. A. (2011). Slow $\dot{V}$O_2_ kinetics during moderate‐intensity exercise as markers of lower metabolic stability and lower exercise tolerance. European Journal of Applied Physiology, 111, 345–355. doi:10.1007/s00421-010-1609-1 20821336

[phy215361-bib-0013] Grassi, B. , Rossiter, H. B. , & Zoladz, J. A. (2015). Skeletal muscle fatigue and decreased efficiency: Two sides of the same coin? Exercise and Sport Sciences Reviews, 43, 75–83. 25688762 10.1249/JES.0000000000000043

[phy215361-bib-0014] Guignard, B. , Rouard, A. , Chollet, D. , Ayad, O. , Bonifazi, M. , Vedova, D. D. , & Seifert, L. (2017). Perception and action in swimming: Effects of aquatic environment on upper limb inter‐segmental coordination. Human Movement Science, 55, 240–254. 10.1016/j.humov.2017.08.003 28846856

[phy215361-bib-0015] Holmér, I. (1972). Oxygen uptake during swimming in man. Journal of Applied Physiology, 33(4), 502–509. 5075849 10.1152/jappl.1972.33.4.502

[phy215361-bib-0016] Hopkins, W. G. , Marshall, S. W. , Batterham, A. M. , & Hanin, J. (2009). Progressive statistics for studies in sports medicine and exercise science. Medicine & Science in Sports & Exercise, 41, 3–12. 19092709 10.1249/MSS.0b013e31818cb278

[phy215361-bib-0017] Jones, A. M. , Grassi, B. , Christensen, P. M. , Krustrup, P. , Bangsbo, J. , & Poole, D. C. (2011). Slow component of $\dot{V}$O_2_ kinetics: Mechanistic bases and practical applications. Medicine & Science in Sports & Exercise, 43(11), 2046–2062. 10.1249/MSS.0b013e31821fcfc1 21552162

[phy215361-bib-0018] Jones, A. M. , & Poole, D. C. (2005a). Oxygen uptake dynamics: From muscle to mouth‐an introduction to the symposium. Medicine & Science in Sports & Exercise, 37(9), 1542–1550. 10.1249/01.mss0000177466.01232.7e 16177607

[phy215361-bib-0019] Jones, A. M. , & Poole, D. C. (2005b). Oxygen uptake kinetics in sport, exercise and medicine. Routledge.

[phy215361-bib-0020] Lisker, G. , Greenberg, H. , Lisker, J. , & Korotun, M. (2020). End tidal CO_2_ levels in healthy adults while breathing through a full‐face snorkel mask. Chest, 157(6S), 378A.

[phy215361-bib-0021] Lomax, M. , Mayger, B. , Saynor, Z. L. , Vine, C. , & Massey, H. C. (2019). Practical considerations for assessing pulmonary gas exchange and ventilation during flume swimming using the MetaSwim metabolic cart. Journal of Strength and Conditioning Research, 33, 1941–1953. 10.1519/JSC.0000000000002801 30113916

[phy215361-bib-0022] Maglischo, E. W. (1993). Swimming even faster. Mayfield Publishing.

[phy215361-bib-0023] McParland, C. , Mink, J. , & Gallagher, C. G. (1991). Respiratory adaptations to dead space loading during maximal incremental loading. Journal of Applied Physiology, 70, 55–62. 2010409 10.1152/jappl.1991.70.1.55

[phy215361-bib-0024] Nuber, G. W. , Jobe, F. W. , Perry, J. , Moynes, D. R. , & Antonelli, D. (1986). Fine wire electromyography analysis of muscles of the shoulder during swimming. American Journal of Sports Medicine, 14, 7–11. 3752349 10.1177/036354658601400102

[phy215361-bib-0025] Oliveira, D. R. , Goncalves, L. F. , Reis, A. M. , Fernandes, R. J. , Garrido, N. D. , & Reis, V. M. (2016). The oxygen uptake slow component at submaximal intensities in breaststroke swimming. Journal of Human Kinetics, 51, 165–173. 28149379 10.1515/hukin-2015-0179 PMC5260559

[phy215361-bib-0026] Pelarigo, J. G. , Machado, L. , Fernandes, R. J. , Greco, C. C. , & Vilas‐Boas, J. P. (2017). Oxygen uptake kinetics and exergy system's contribution around maximal lactate steady state swimming intensity. PLoS One, 12(2), e0167263. 10.1371/journal.pone.0167263 28245246 PMC5330462

[phy215361-bib-0027] Pringle, J. S. , Doust, J. H. , Carter, H. , Tolfrey, K. , Campbell, I. T. , & Jones, A. M. (2003). Oxygen uptake kinetics during moderate, heavy and severe intensity ‘submaximal’ exercise in humans: The influence of muscle fibre type and capilliarisation. European Journal of Applied Physiology, 89, 289–300. 10.1007/s00421-003-0799-1 12736837

[phy215361-bib-0028] Reis, J. F. , Alves, F. B. , Bruno, P. M. , Vleck, V. , & Millet, G. P. (2012a). Effects of aerobic fitness on oxygen uptake kinetics in heavy intensity swimming. European Journal of Applied Physiology, 112, 1689–1697. 10.1007/s00421-011-2126-6 21879352

[phy215361-bib-0029] Reis, J. F. , Alves, F. B. , Bruno, P. M. , Vleck, V. , & Millet, G. P. (2012b). Oxygen uptake kinetics and middle distance swimming performance. Journal of Science and Medicine in Sport, 15, 58–63. 10.1016/j.jsams.2011.05.12 21802360

[phy215361-bib-0030] Reis, J. F. , Millet, G. P. , Bruno, P. M. , Vleck, V. , & Alves, F. B. (2017). Sex and exercise intensity do not influence oxygen uptake kinetics in submaximal swimming. Frontiers in Physiology, 8(72), 1–8. 10.3389/fphys.2017.00072 28239356 PMC5301027

[phy215361-bib-0031] Sousa, A. , Jesus, K. D. , Figueiredo, P. , Vilas‐Boas, J. P. , & Fernandes, R. J. (2013). Oxygen uptake kinetics at moderate and extreme swimming intensities. Revista Brasileira de Medicina do Esporte, 19(3), 186–190.

[phy215361-bib-0032] Sousa, A. C. , Vilas‐Boas, J. P. , & Fernandes, R. J. (2014). VO_2_ kinetics and metabolic contributions whilst swimming at 95, 100m, and 105% of the velocity at VO_2max_ . BioMed Research International, 2014(675363), 1–9. 10.1155/2014/675363.ff PMC4087294 25045690

[phy215361-bib-0033] Ward, S. , & Whipp, B. J. (1980). Ventilatory control during exercise with increased external dead space. Journal of Applied Physiology, 48, 225–231. 6767666 10.1152/jappl.1980.48.2.225

[phy215361-bib-0034] Wilson, B. D. , Takagi, H. , & Pease, D. P. (1998). Technique comparison of pool and flume swimming. In K. L. Keskinen , P. V. Komi , & A. P. Hollander (Eds.), Scientific Proceedings of the VIIIth International Symposium of Biomechanics and Medicine in Swimming (BMS) (pp. 181–184). University of Jyväskylä.

